# Integrating Traditional Nutritional Wisdom into Digital Nutrition Platforms: Toward Culturally Adaptive and Inclusive Health Technologies

**DOI:** 10.3390/nu17121978

**Published:** 2025-06-11

**Authors:** Camila Suarez, Sasan Adibi

**Affiliations:** 1 Department of Food and Health, University of Padova, 35122 Padova, Italy; 2 School of IT, Monash University, Clayton, VIC 3800, Australia; sasan.adibi@monash.edu

**Keywords:** digital nutrition, traditional food systems, cultural adaptation, personalized nutrition, eHealth, nutrition informatics, health equity, Indigenous knowledge, user-centred design, nutrition technology, digital storytelling, generative AI, cultural sustainability, participatory methods, knowledge translation

## Abstract

**Background/Objectives:** Traditional nutritional knowledge, shaped by centuries of cultural and ecological adaptation, offers holistic and sustainable dietary frameworks that remain highly relevant to modern health challenges. However, current digital nutrition platforms often fail to reflect this diversity, relying instead on standardized models with limited cultural sensitivity. This paper aims to explore how traditional nutritional wisdom can be integrated into digital health platforms to promote more inclusive and effective approaches to personalized nutrition. **Methods:** This perspective paper employs a cultural adaptation framework to analyze the integration of traditional food knowledge into digital contexts. Drawing from interdisciplinary research across nutrition science, anthropology, digital health and implementation science, we utilize the Knowledge-to-Action (KTA) Framework and the PEN-3 Cultural Model to structure our analysis. A systematic scoping review of literature published between 2010 and 2025 was conducted to identify integration challenges and opportunities. Additionally, we analyzed case studies of three traditional dietary systems (Argentina, Italy and Japan) and evaluated five leading digital nutrition platforms for their degree of cultural inclusivity, using qualitative comparative methods. **Results:** The analysis highlights significant challenges in adapting traditional knowledge to digital formats, including standardization barriers, contextual loss and technological limitations. However, successful integration initiatives demonstrate that through participatory design, flexible data architectures and culturally-informed algorithms, traditional food systems can be meaningfully represented. Our proposed four-phase integration framework—documentation, digital adaptation, implementation and evaluation—provides a structured approach for developers and researchers. **Conclusions:** Bridging traditional nutrition with digital platforms represents a vital opportunity to enhance personalization and preserve food heritage while improving health outcomes for diverse populations. This integration requires interdisciplinary collaboration, user-centered design processes and ethical approaches that respect cultural ownership and context.

## 1. Introduction

Across continents and centuries, traditional nutritional wisdom has played a central role in shaping community health, cultural identity and ecological balance. These systems, developed through lived experience and environmental adaptation, remain a vital source of insight into sustainable and health-promoting dietary patterns. From the energetic principles of traditional Chinese and Ayurvedic nutrition to the seasonal and communal ethos of the Mediterranean diet [1,2,3], such practices have long understood food not only as nourishment, but as a source of healing, connection and harmony with nature.

Meanwhile, digital health platforms have rapidly emerged as transformative tools in healthcare delivery, including the field of nutrition [4]. Mobile apps, wearables and AI-powered dietary systems now guide how millions manage their eating habits, monitor health indicators and receive nutritional advice. However, many of these platforms are built upon standardized Western models that often overlook cultural nuance, food heritage and the deeply contextual nature of traditional food systems [5,6]. This raises concerns about inclusivity, cultural erosion and the potential misalignment between technological innovation and diverse human experiences.

To address these challenges, this paper draws upon two complementary theoretical frameworks. The Knowledge-to-Action (KTA) Framework guides the process of translating traditional nutritional knowledge—often embedded in oral, ritual and community-based practices—into actionable formats suitable for digital platforms [7]. Simultaneously, the PEN-3 Cultural Model developed in the context of public health, supports the cultural grounding of interventions by centering community perceptions, enablers and nurturers [8]. By integrating these models, we aim to bridge tradition and technology in ways that honor cultural diversity while enhancing digital innovation.

This perspective paper examines culturally rooted food systems—drawing on examples from Argentina, Italy and Japan—and explores how they can be adapted into digital formats in ways that preserve their richness and relevance. By identifying current limitations and proposing a culturally adaptive framework for integration. The paper aims to contribute to a more inclusive, ethical and responsive future for digital nutrition innovation.

By addressing this integration challenge through the lens of implementation science and cultural adaptation theory [8,9], we aim to contribute practical insights for researchers, developers and health practitioners working at the intersection of traditional nutrition knowledge and digital innovation. Our specific objectives are to (1) analyze the key components of traditional food systems that offer value for digital integration; (2) identify technical and cultural barriers to successful integration; (3) evaluate existing integration approaches through case studies; and (4) propose a comprehensive framework for ethically incorporating traditional nutritional wisdom into digital platforms.

## 2. Theoretical Framework and Methods

### 2.1. Theoretical Approach

This paper employs cultural adaptation theory [8] and the Consolidated Framework for Implementation Research (CFIR) [9] to analyze the integration of traditional nutritional knowledge into digital contexts. Cultural adaptation theory provides a structured approach to understanding how culturally grounded practices can be respectfully translated across contexts while maintaining core elements and values. The CFIR offers a comprehensive framework for identifying factors that influence the implementation of innovations across different settings, making it particularly useful for understanding the complexities of integrating traditional knowledge into technology-based interventions.

We specifically apply Bernal’s cultural adaptation model [10], which emphasizes eight dimensions of adaptation: language, persons, metaphors, content, concepts, goals, methods and context. This framework helps structure our analysis of how traditional nutritional elements can be appropriately translated into digital formats while preserving their cultural integrity and effectiveness.

### 2.2. Methods

This perspective synthesis was conducted through the following:

1. **Systematic literature review**: We searched the PubMed, Scopus and Web of Science databases for studies published between 2010 and 2025 using combinations of terms related to traditional nutrition, digital health, cultural adaptation and implementation science. Articles were screened for relevance to the integration of traditional dietary knowledge into modern health systems or digital platforms.
2. **Case study analysis**: We selected three diverse traditional food systems (Argentina, Italy and Japan) based on their geographical distribution, documentation quality and distinctive nutritional approaches. For each system, we analyzed key principles, practices and potential elements for digital integration.
3. **Digital platform evaluation**: We assessed five leading digital nutrition platforms (MyFitnessPal, Cronometer, Noom, Lifesum and Nutritics) using a cultural inclusivity framework adapted from Montague and Perchonok [11]. Platforms were evaluated based on representation of diverse foods, cultural adaptability, contextual information and integration of traditional dietary concepts.
4. **Framework development**: Based on the literature review, case studies and platform evaluations, we developed an integration framework that was then refined through consultation with experts in digital health, cultural anthropology and traditional nutrition systems.

This methodological approach allows for a comprehensive analysis of both the challenges and opportunities in digitizing traditional nutritional knowledge while remaining grounded in established theoretical frameworks for cultural adaptation and implementation.

## 3. Background

### 3.1. Overview of Traditional Nutritional Systems Worldwide

Traditional nutritional systems reflect centuries of accumulated knowledge shaped by environment, culture and health beliefs [12]. While diverse in form and philosophy, these systems often emphasize seasonal eating, whole foods, communal practices and a holistic approach to well-being.

#### 3.1.1. Argentina

In Argentina, traditional nutrition has been strongly influenced by Indigenous practices, European colonization (especially Spanish and Italian) and rural food culture [13]. Staples include beef, maíz (corn), yerba mate and vegetables like squash and sweet potatoes. Historically, Indigenous groups such as the Mapuche and Guaraní maintained diets based on local grains, legumes and wild plants, often incorporating herbal medicine into daily life [14]. While modern Argentine cuisine is heavily meat-based, the traditional use of mate (an herbal infusion) continues to play a central role in digestion and social connection [15].

#### 3.1.2. Italy

The Mediterranean diet, recognized by UNESCO as an intangible cultural heritage, originates in part from southern Italy and emphasizes olive oil, whole grains, fresh vegetables, legumes, fish and moderate wine consumption [16]. Beyond nutrients, this dietary pattern values communal meals, slow eating and a strong connection to regional, seasonal foods. Scientific research supports its role in reducing cardiovascular risk and inflammation [17]. Italian food wisdom also includes fermentation (e.g., cheeses) and a “non-industrial” food philosophy that contrasts with modern fast-food culture [18].

#### 3.1.3. Japan

Traditional Japanese nutrition is rooted in the principles of balance, simplicity and seasonal harmony. The traditional diet, often referred to as *washoku* (*Japanese cuisine*), includes rice, miso soup, fish, seaweed, fermented vegetables and green tea [19]. Meals are typically built on the principle of *ichiju-sansai* (*one soup, three sides*), promoting variety and portion control. Fermented foods like natto, miso and *tsukemono* (*Japanese pickles*) contribute to gut health and immune support, while the inclusion of seaweed provides minerals like iodine and calcium [20]. The Japanese diet also emphasizes *umami*, the “fifth taste,” which enhances flavor without relying on excess salt or fat. Importantly, mindful eating practices—such as expressing gratitude (*itadakimasu*)—reinforce the connection between food, respect and well-being [21].

This nutritional model is associated with one of the highest life expectancies in the world and Japan has among the lowest obesity rates globally. Despite increasing Westernization of diets in recent decades, traditional food culture remains deeply respected and is being actively revived for its health and longevity benefits [22].

### 3.2. Historical Context and Evolution of Traditional Food Wisdom

Before the rise of industrialized agriculture and processed foods, most communities relied on locally sourced and minimally processed ingredients. Food was selected and prepared based on climate, availability, tradition and often spiritual or medicinal beliefs. This knowledge was passed down orally or through cultural rituals and embedded in daily life [23].

Globalization and modernization led to a significant erosion of traditional food systems [24]. In many regions, Western dietary patterns replaced Indigenous diets, contributing to rising rates of chronic diseases [25]. Simultaneously, the disconnect from land, food origin and ancestral practices weakened community resilience and cultural identity [14]. Today, there is a growing global movement to revive and preserve traditional dietary knowledge, not only for cultural preservation but also for its relevance to sustainability and food system resilience [26].

Traditional food wisdom is deeply intertwined with the cultural, spiritual and ecological life of communities. These practices are not merely culinary but are embedded in ceremonies and everyday rituals that transmit values, identity and belonging. In Argentina, for instance, the preparation of *locro*—a hearty stew traditionally consumed on national holidays—carries Indigenous and colonial legacies, combining local ingredients with symbolic meaning tied to national identity and seasonal celebration [27]. The communal sharing of *mate*, beyond its nutritional content, serves as a social ritual and act of hospitality across generations [27].

In Japan, the traditional dietary practice of *washoku* exemplifies food as philosophy, emphasizing balance, seasonality and aesthetic harmony. The *ichiju-sansai* principle (one soup, three side dishes) promotes dietary diversity and portion control, while rituals like *itadakimasu* and *gochisousama* reinforce gratitude and mindfulness. These traditions, recognized by UNESCO as intangible cultural heritage, are kept alive through intergenerational transmission and community education programs [28].

Italy offers another case study of historical resilience and adaptation. During periods of scarcity in post-war southern Italy, rural communities relied on *cucina povera* (*peasant cuisine*), creating nutritionally dense meals from legumes, stale bread, wild herbs and local vegetables [29]. Dishes like *ribollita* (*reboiled*) or *minestra di ceci* (*chickpea soup*) emerged as sustainable solutions, preserving nutritional quality with minimal resources—practices that align with today’s goals for low-impact diets.

These examples demonstrate how traditional food systems have adapted to shifting environments, technologies and socio-political pressures without losing their core values. They are dynamic, living systems shaped by resilience and innovation—not static relics of the past [30]. By incorporating such rich contextual histories into digital platforms, we can ensure that technology respects the fluid and culturally anchored nature of traditional food knowledge.

### 3.3. Current State of Digital Health Platforms in Nutrition

While digital health platforms have made strides in data collection and behavior tracking, they often lack alignment with culturally specific nutrition frameworks. Bridging this gap is essential to ensure equity in digital nutrition and harness the full potential of nutrition informatics [27].

Over the past decade, digital health platforms have revolutionized how individuals track, monitor and manage their nutritional habits. These include the following:

- Mobile apps for calorie counting, meal planning and fasting
- Wearables that track activity and energy expenditure
- AI-driven tools for personalized diet recommendations
- Tele-nutrition platforms for remote dietitian consultations

However, most of these tools are developed with a standardized Western-centric nutritional model, often based on calorie control, macronutrient ratios and BMI tracking. They tend to lack integration of culturally specific foods, traditional eating patterns and non-quantitative factors like mealtime rituals, emotional connection to food or ancestral wisdom [28].

As a result, current platforms risk oversimplifying nutrition and excluding diverse populations whose health practices don’t align with mainstream models. There is a pressing need for digital health tools that are inclusive and culturally adaptable to the diverse ways people engage with food around the world [29].

Our analysis of five major nutrition platforms (Table 1) reveals significant limitations in cultural inclusivity and traditional knowledge integration.

Our evaluation found that while some platforms (particularly Cronometer and Nutritics) offer relatively diverse food databases, all platforms demonstrated significant limitations in representing cultural context, traditional nutritional principles and adaptability to diverse food systems. Common issues include the following:

- Standardization around Western nutritional paradigms (calories, macronutrients)
- Limited representation of traditional preparation methods and their nutritional impacts
- Absence of seasonal, regional, or cultural context for food choices
- Minimal integration of traditional wisdom about food combinations, timing, or energetic properties
- Limited adaptability to the diverse cultural understanding of portion sizes and meal structures

These limitations represent missed opportunities for delivering truly personalized nutrition guidance that resonates with users’ cultural backgrounds and food traditions. As digital health tools continue to evolve, addressing these gaps becomes increasingly important for both inclusivity and effectiveness.

## 4. The Value of Traditional Nutritional Knowledge

### 4.1. Evidence-Based Benefits of Traditional Dietary Patterns

Scientific research has increasingly validated the health benefits of traditional dietary patterns [30]. A growing body of evidence from epidemiological studies, clinical trials and systematic reviews demonstrates that diets rooted in ancestral wisdom often promote better health outcomes than modern Western dietary patterns.

The Mediterranean diet has been extensively studied, with multiple randomized controlled trials demonstrating its effectiveness in reducing cardiovascular disease risk. The landmark PREDIMED study found that adherence to a Mediterranean diet supplemented with extra-virgin olive oil or nuts reduced major cardiovascular events by approximately 30% compared to a low-fat control diet [31]. Meta-analyses have confirmed these benefits, showing significant reductions in overall mortality, cardiovascular mortality, cancer incidence and neurodegenerative diseases [32].

Similarly, the traditional Japanese diet has been associated with remarkable longevity and metabolic health. The Japan Public Health Center-based Prospective Study, which followed over 79,000 participants for an average of 15 years, found that higher adherence to the traditional Japanese dietary guidelines was associated with a 15% lower mortality rate [33]. The Okinawan diet in particular—characterized by sweet potatoes, vegetables, soy products and moderate caloric intake—has been linked to exceptional longevity and low rates of age-related diseases [34].

In Latin America, traditional plant-based diets rich in beans, corn and native plants have shown protective effects against metabolic syndrome and its components. A systematic review of traditional Latin American diets found consistent associations between adherence to traditional dietary patterns and lower rates of obesity, hypertension and diabetes compared to Western dietary patterns [35].

These scientific findings validate what traditional food cultures have maintained through generations of observation and experience. While these dietary patterns vary substantially in their specific components, they share common elements that appear to promote health: emphasis on plant foods, moderate protein intake, minimal processing and strong social/cultural food contexts.

### 4.2. Cultural Significance and Sustainable Aspects

Traditional food systems are deeply embedded in cultural identity, social rituals and ecological balance. Unlike standardized nutritional frameworks that often isolate food from its context, traditional dietary wisdom reflects a holistic understanding of nourishment—one that includes symbolic meaning, seasonal rhythms and intergenerational transmission of knowledge [36].

#### 4.2.1. Cultural Significance

While the historical evolution of traditional food wisdom highlights how communities have preserved and adapted their culinary practices over time, cultural significance emphasizes how these practices continue to shape contemporary identities, social cohesion and mental well-being.

Traditional food rituals strengthen bonds within families and communities, particularly through shared preparation, storytelling and seasonal gatherings. These moments act as a form of intergenerational learning and identity-building, often becoming the glue that connects diaspora populations to their heritage [37]. For example, younger generations learning to prepare *locro* or *ribollita* (*reboiled*) not only acquire cooking skills but also reconnect with values such as reciprocity, resilience and care.

Furthermore, the symbolic dimensions of food rituals contribute to emotional and psychological health. Practices like *itadakimasu* (*expressing gratitude*) in Japan or the sharing of *mate* in Argentina promote mindfulness, gratitude and mutual respect—elements increasingly recognized as essential to holistic well-being [38]. Cultural food practices also offer safe spaces for affirming marginalized identities, particularly among Indigenous and migrant communities whose dietary customs are often misrepresented or excluded from dominant narratives [39].

By recognizing the non-nutritional value of food—its role in memory, identity, healing and resistance—we ensure that digital platforms designed to support health do not overlook the deeper meanings embedded in traditional food systems.

#### 4.2.2. Sustainable Aspects

Traditional food systems often embody principles of sustainability long before the term became mainstream in global policy. Rooted in local ecologies and knowledge of natural cycles, these practices prioritize seasonal availability, biodiversity, low waste and community resilience [40].

For example, the Mediterranean and Andean diets rely on time-tested agricultural methods that maintain soil fertility, support crop rotation and minimize dependence on chemical inputs [41]. In many rural Italian regions, recipes like minestra di ceci emerged from cucina povera traditions that reduced food waste by repurposing leftovers and promoting full use of available ingredients [42]. Similarly, Indigenous farming systems in Latin America—such as the milpa cycle of maize, beans and squash—sustain biodiversity while meeting nutritional needs with minimal environmental impact [42].

In Japan, the washoku approach encourages balanced, plant-forward meals with modest portions of animal products, aligning with modern planetary health guidelines. The emphasis on fermentation not only supports gut health but also extends shelf life without relying on artificial preservatives or refrigeration. These low-impact preservation techniques illustrate how traditional knowledge can contribute to carbon footprint reduction and food security [41].

Moreover, traditional diets are inherently adapted to climate and geography, offering valuable insights for sustainable dietary transitions in response to environmental change. Reviving and digitizing this wisdom—especially when embedded in platforms that promote local, seasonal choices—can support efforts to reduce food miles, cut emissions and foster more resilient food systems globally [43].

By integrating these sustainable logics into digital nutrition tools, we can move beyond individual nutrition optimization to embrace ecological and community-centered well-being, aligning digital innovation with planetary health values.

### 4.3. Holistic Approaches to Nutrition in Traditional Systems

Unlike many modern approaches that reduce food to its nutrient components, traditional nutritional systems often take a holistic view of health, considering the relationships between mind, body, environment and sometimes spirituality. This integrated perspective offers insights that complement contemporary nutritional science in important ways.

In Ayurvedic and Traditional Chinese Medicine (TCM), for instance, food is tailored to individual constitutions (doshas or yin-yang balance), seasons and emotional states. These systems recognize the importance of digestive capacity, timing of meals and the energetic qualities of food, which are often overlooked in calorie-focused models [44,45]. Recent research in chronobiology and personalized nutrition has begun to validate some of these traditional concepts, showing that identical meals can produce different metabolic effects based on timing, individual differences and prior meal patterns [46].

In Japan, the principle of hara hachi bu—eating until 80% full—reflects mindfulness and respect for the body’s natural hunger cues [47]. This practice has been linked to metabolic health and longevity through pathways involving reduced oxidative stress and improved cellular maintenance [48]. Similarly, in many Latin American indigenous communities, food preparation involves storytelling and communal participation, supporting mental and emotional well-being alongside nutrition—an approach that aligns with emerging research on the psychological dimensions of eating [49].

These holistic elements of traditional food systems—attention to individuality, seasonality, mindfulness and food’s emotional and social dimensions—represent valuable complements to the nutrient-focused approach that dominates conventional nutritional science and most digital nutrition platforms. Integrating these elements could significantly enhance the effectiveness and relevance of digital nutrition guidance.

## 5. Challenges in Digital Integration

### 5.1. Cultural Representation and Standardization Issues

The translation of traditional food knowledge into digital formats faces significant challenges related to cultural representation and standardization. Traditional nutritional systems are inherently diverse and contextual and often resist the standardization required by digital platforms.

One fundamental issue is the categorization and naming of traditional foods. Digital platforms typically require standardized food databases, but traditional dishes often have regional variations, multiple names, or preparation methods that significantly affect their nutritional composition. For example, the Argentine dish “locro” varies substantially across regions, with variations in ingredients and preparation methods that affect both its nutrient profile and cultural significance [50]. Similarly, Japanese fermented foods like natto have properties that change based on fermentation duration and techniques that are difficult to capture in standardized databases [51].

Even more challenging is representing the non-quantitative aspects of traditional food systems. Many traditional approaches classify foods based on qualities that don’t neatly map to nutrition database fields—“warming” versus “cooling” foods in TCM, the Ayurvedic classification of foods by taste (rasa) and post-digestive effect (vipaka), or the Japanese concept of food as medicine (yakuzen) [52]. These concepts are central to how these systems guide food choices but have no direct equivalents in conventional nutrition data models.

The risk of misrepresentation is substantial when traditional foods are forced into standardized frameworks. A study analyzing the representation of traditional African foods in international nutrition databases found error rates of up to 30% in nutrient values, with many traditional preparation methods either missing entirely or significantly mischaracterized [53]. This misrepresentation can lead to both cultural loss and inaccurate nutritional guidance.

One potential solution is to develop flexible, culturally adaptive database structures, such as semantic web technologies or graph databases, that allow for multiple names, preparation variations, symbolic meanings and regional differences to be properly represented alongside nutritional profiles.

### 5.2. Technical Challenges in Digitizing Traditional Knowledge

Beyond cultural representation, specific technical challenges arise when attempting to digitize traditional nutritional knowledge. These technical barriers exist at multiple levels, from data structure to algorithm design.

#### 5.2.1. Data Architecture Limitations

Most digital nutrition platforms use relational database structures that organize foods hierarchically by category and nutrient content. These structures struggle to represent the following:

- Foods with highly variable composition (e.g., foraged plants or traditional ferments)
- Context-dependent food qualities (e.g., seasonal variations, preparation methods)
- Relational aspects of foods (e.g., traditional combinations or sequential consumption)
- Cultural metadata that provides meaning beyond nutrition

A review of 20 nutrition database architectures found that none adequately accommodated the complex, relational nature of traditional food systems [54]. Alternative data structures such as graph databases or semantic web approaches may better capture the interconnected nature of traditional food knowledge but require significant development investment.

#### 5.2.2. Algorithm Design Challenges

The algorithms that power personalized nutrition recommendations in digital platforms typically rely on optimization around a limited set of parameters (e.g., caloric targets, macronutrient distributions). These approaches struggle to incorporate traditional nutritional wisdom that might prioritize the following:

- Energetic balance over macronutrient ratios
- Seasonal appropriateness over year-round availability
- Cultural context over individual preference
- Traditional food combinations over isolated nutrient optimization

Implementing algorithms that respect these traditional principles requires not only different technical approaches but also different epistemological frameworks that value traditional ways of knowing alongside scientific evidence [55]. Machine learning approaches that incorporate multiple knowledge systems show promise but remain in the early development stages for nutrition applications [56].

#### 5.2.3. User Interface and Experience Barriers

User interfaces designed around Western nutritional concepts may feel foreign or irrelevant to those practicing traditional dietary approaches. Common interface elements in nutrition apps—calorie counters, macronutrient trackers, or food logging interfaces organized by Western meal patterns—may not align with traditional ways of conceptualizing or interacting with food.

Research on cultural usability in digital health tools has identified significant barriers when interfaces do not reflect users’ cultural models of health and well-being [57]. For traditional food systems, these barriers can be particularly pronounced when interfaces the following:

- Present food primarily as a collection of nutrients rather than as culturally meaningful entities
- Organize meal planning around Western meal patterns (breakfast, lunch, dinner)
- Use visualization approaches that emphasize quantitative rather than qualitative aspects
- Exclude important contextual elements like eating environment, food preparation rituals, or social dimensions

These technical challenges highlight the need for a fundamental rethinking of how digital nutrition platforms are designed, from data architecture to algorithm design to user experience. Addressing these challenges requires not only technical innovation but also interdisciplinary collaboration between technologists, cultural anthropologists, nutrition scientists and representatives of traditional food cultures.

One potential solution is to adopt participatory design approaches, involving traditional knowledge holders in co-designing database structures, algorithm logic and culturally appropriate interface models, ensuring systems are flexible enough to reflect diverse and context-sensitive food systems.

### 5.3. Ethical Concerns and Knowledge Protection

The digitization of traditional food knowledge raises significant ethical concerns around appropriation, misrepresentation and intellectual property rights. These concerns extend beyond technical challenges to fundamental questions about power, cultural sovereignty and respect.

Traditional food knowledge often originates from Indigenous and local communities who have historically faced exploitation and marginalization. Digital platforms that incorporate this knowledge without proper attribution, consent, or benefit-sharing risk perpetuating these patterns of exploitation [58]. This is particularly concerning when traditional knowledge is commercialized without acknowledgment or compensation to the communities of origin.

Several international frameworks address the protection of traditional knowledge, including the United Nations Declaration on the Rights of Indigenous Peoples and the Nagoya Protocol on Access and Benefit-Sharing [59]. However, these frameworks often have limited applicability to digital contexts, creating uncertainty around appropriate governance for digitized traditional knowledge.

A 2023 survey of digital health applications incorporating traditional knowledge found that only 17% had explicit policies for attribution and benefit-sharing, while just 12% involved representative community participation in their development [60]. This suggests that ethical considerations remain underdeveloped in most digital nutrition platforms that incorporate traditional elements.

Key ethical challenges include the following:

- Obtaining meaningful informed consent from knowledge holders who may have different conceptions of knowledge ownership
- Ensuring appropriate attribution that respects both individual contributors and collective traditions
- Developing benefit-sharing mechanisms that recognize the communal nature of traditional knowledge
- Protecting knowledge that communities may wish to keep private or share only in specific contexts
- Balancing open access principles with respect for cultural protocols around knowledge sharing

One potential solution is to embed ethical frameworks like the CARE Principles (Collective Benefit, Authority to Control, Responsibility and Ethics) directly into platform design, coupled with participatory governance models that allow communities to retain authority over how their knowledge is shared, used and credited.

Addressing these ethical concerns requires both technical solutions (such as digital rights management systems) and governance approaches that center the perspectives and interests of traditional knowledge holders [61]. Participatory design methodologies, community approval processes and transparent benefit-sharing agreements represent important steps toward more ethical integration of traditional food knowledge in digital platforms.

## 6. Existing Models and Case Studies

While the integration of traditional nutritional knowledge into digital platforms remains an emerging field, several promising initiatives demonstrate the potential for respectful and effective digital adaptation. These case studies illustrate diverse approaches to bridging traditional wisdom and digital innovation.

### 6.1. Community-Led Digital Documentation Initiatives

The Traditional Food Systems Archive (TFSA) in Peru represents a model of community-controlled digital documentation [62]. Initiated by Indigenous communities in collaboration with nutritional anthropologists, this digital archive preserves traditional Andean food knowledge through multimedia documentation, including the following:

- Video recordings of traditional preparation techniques
- Seasonal calendars for wild and cultivated foods
- Oral histories about food-related traditions and ceremonies
- Detailed information about traditional food combinations and their purposes

Critically, the TFSA employs a community governance model where decisions about what knowledge to digitize, how to represent it and who can access it remain with the elder councils of participating communities. The platform uses a custom ontology developed collaboratively with community knowledge holders to organize information in culturally appropriate ways that preserve relational aspects of traditional knowledge.

Evaluation research conducted with both community members and external users indicates high levels of cultural authenticity and usability [63]. Community members reported strong satisfaction with how their traditions were represented (92% approval rating), while nutritionists and researchers noted the platform’s ability to convey contextual information missing from conventional nutrition databases.

### 6.2. Culturally Adapted Commercial Applications

Several commercial nutrition applications have begun incorporating elements of traditional food systems, though with varying levels of cultural depth and community involvement.

The Japanese Washoku Balance app combines traditional Japanese dietary principles with modern nutrition tracking [64]. Developed by nutritionists and washoku experts, the application

- Organizes meal planning around the traditional ichiju-sansai structure
- Includes seasonal food recommendations based on the traditional Japanese calendar
- Provides guidance on traditional food preparation techniques
- Incorporates traditional wisdom about food balance and portion control

The app has shown promising results in pilot studies, with users reporting higher adherence to traditional dietary patterns and greater cultural connectedness compared to conventional calorie-tracking apps [65]. However, critics note that the commercial nature of the platform limits community governance and may simplify complex cultural traditions.

In contrast, the Mediterranean Diet App Consortium (MyFitnessPal app—25.22.0) represents a multi-stakeholder approach involving research institutions, community organizations and technology companies across Mediterranean countries [66]. This initiative has developed open-source tools and standards for representing Mediterranean dietary traditions in digital formats, including the following:

- A collaborative food database reflecting regional variations in traditional dishes
- Cultural context modules that explain the history and significance of food practices
- Meal pattern templates that respect traditional eating rhythms and social dimensions
- Open API standards that allow diverse applications to incorporate these elements

This consortium approach enables multiple applications to incorporate authentic cultural elements while maintaining community involvement in knowledge governance. However, challenges remain in sustainable funding and balancing cultural specificity with user-friendly design.

### 6.3. Integration with Health Systems and Research Platforms

The Indigenous Health Adaptation to Climate Change (IHACC) project demonstrates how traditional food knowledge can be integrated with formal health systems through digital tools [67]. Working with communities in Peru, Uganda and Canada, this initiative has developed digital systems that

- Document relationships between traditional food practices and health outcomes;
- Combine Indigenous environmental knowledge with climate data to predict food availability;
- Support community-led monitoring of food system changes;
- Facilitate knowledge exchange between traditional healers and healthcare providers.

A key innovation of the IHACC platform is its multi-ontology architecture, which maintains both Indigenous and scientific knowledge systems in parallel rather than forcing traditional knowledge into scientific frameworks [68]. This approach preserves the integrity of traditional knowledge while enabling interoperability with conventional health information systems.

Evaluation of this approach in northern Canada found that it effectively supported the integration of traditional food recommendations into primary healthcare, with 78% of participating clinicians reporting improved understanding of traditional practices and 64% reporting changes in their nutritional counseling approaches [69].

### 6.4. Key Success Factors and Lessons Learned

Analysis of these case studies reveals several common factors associated with the successful integration of traditional nutritional knowledge into digital platforms:

1. **Community governance and participation**: Successful initiatives involve knowledge-holding communities at all stages, from conceptualization to implementation and evaluation.
2. **Flexible data architectures**: Rather than forcing traditional knowledge into conventional database structures, effective platforms employ flexible architectures that can represent diverse knowledge systems.
3. **Multimedia documentation**: Beyond text and numbers, successful platforms incorporate visual, audio and interactive elements that better capture the multidimensional nature of traditional knowledge.
4. **Contextual preservation**: Effective platforms maintain connections between foods, practices, cultural contexts and underlying values rather than isolating nutritional components.
5. **Formal recognition of knowledge sources**: Successful initiatives implement clear attribution and benefit-sharing mechanisms that acknowledge the origins of traditional knowledge.
6. **Interoperability with conventional systems**: While maintaining cultural integrity, effective platforms also enable dialogue with conventional nutrition and healthcare systems.

These success factors provide valuable guidance for the development of more comprehensive approaches to integrating traditional nutritional wisdom into digital platforms, as outlined in the next section.

## 7. Proposed Framework for Integration

Building on the theoretical foundations, challenges and successful models identified in previous sections, we propose a comprehensive framework for integrating traditional nutritional wisdom into digital platforms. This framework addresses both technical and cultural dimensions of integration, providing practical guidance for developers, researchers and communities.

### 7.1. A Four-Phase Integration Model

**Figure 1:**
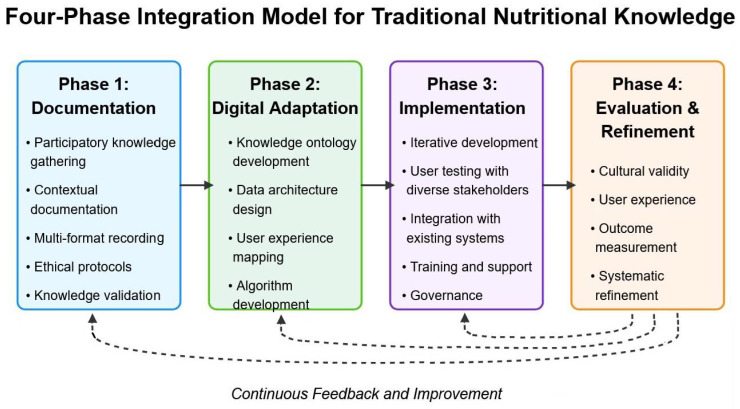
The Four-Phase Integration Model for Traditional Nutritional Knowledge in Digital Platforms.

Our proposed integration framework consists of four interrelated phases: Documentation, Digital Adaptation, Implementation and Evaluation (Figure 1). Each phase incorporates specific processes and principles to ensure cultural integrity, technical feasibility and practical effectiveness.

#### 7.1.1. Phase 1: Documentation

The documentation phase focuses on gathering traditional nutritional knowledge in ways that respect its cultural context and complexity. Key components include the following:

- **Participatory knowledge gathering**: Working with knowledge holders through culturally appropriate methods such as community-based participatory research, oral history interviews and observational documentation.
- **Contextual documentation**: Capturing not just what foods are eaten, but also why, when, how, by whom and in what circumstances, preserving the web of relationships that give traditional foods their meaning.
- **Multi-format recording**: Using diverse documentation formats including video, audio, photography and text to capture dimensions of traditional knowledge that may not translate well to written form.
- **Ethical protocols**: Implementing clear agreements regarding attribution, ownership, privacy, access restrictions and benefit-sharing for all documented knowledge.
- **Knowledge validation**: Engaging community review processes to ensure accuracy and cultural appropriateness of documented information.

This documentation phase establishes the foundation for digital integration by ensuring that traditional knowledge is represented accurately and respectfully before any technical implementation begins.

#### 7.1.2. Phase 2: Digital Adaptation

The digital adaptation phase translates documented knowledge into technical formats that maintain cultural integrity while enabling digital functionality. Key components include the following:

- **Knowledge ontology development**: Creating culturally appropriate knowledge structures that reflect how traditional systems organize and relate food concepts.
- **Data architecture design**: Developing flexible database architectures that can accommodate diverse knowledge representations, including qualitative attributes, contextual variables and relational aspects.
- **User experience mapping**: Designing interface elements and interaction patterns that align with cultural understandings of food and nutrition.
- **Algorithm development**: Creating recommendation and analysis algorithms that incorporate traditional nutritional principles alongside conventional nutritional science.
- **Technical specification creation**: Developing detailed technical requirements and specifications for implementation that preserve cultural nuance.

This phase requires close collaboration between technical experts and cultural knowledge holders to ensure that digital representations remain faithful to traditional understanding while being technically implementable.

To further enhance cultural relevance and engagement, generative AI tools can be employed to enrich digital storytelling and personalization. These technologies allow for the dynamic recreation of oral traditions, community narratives and food preparation rituals—especially valuable where traditional knowledge has been historically transmitted through storytelling rather than written records. For example, AI-driven chatbots can simulate guided cooking sessions with narration styled after community elders, while visual generators can produce culturally authentic imagery of traditional dishes and festive settings.

Generative AI can also support multilingual adaptation, the creation of personalized recommendations based on seasonal and cultural cues, and the visualization of food rituals or ingredient-sourcing practices. When used responsibly and in collaboration with communities, these tools can strengthen user engagement and maintain the narrative continuity that is central to many traditional food systems [70].

#### 7.1.3. Phase 3: Implementation

The implementation phase brings the digitally adapted knowledge into functional platforms accessible to users. Key components include the following:

- **Iterative development**: Employing agile development approaches with regular community feedback cycles to ensure ongoing alignment with cultural expectations.
- **User testing with diverse stakeholders**: Testing with both traditional knowledge holders and potential end-users from diverse backgrounds to identify usability issues and cultural misalignments.
- **Integration with existing systems**: Developing appropriate interfaces with conventional nutrition databases, healthcare systems and other relevant platforms.
- **Training and support development**: Creating resources to help users understand and effectively engage with traditional nutritional concepts.
- **Governance implementation**: Establishing ongoing governance mechanisms for platform management, knowledge updates and issue resolution.

This phase transforms abstract designs into concrete tools while maintaining mechanisms for community oversight and continuing refinement.

#### 7.1.4. Phase 4: Evaluation and Refinement

The evaluation phase assesses both the cultural authenticity and practical effectiveness of the implemented platform. Key components include the following:

- **Cultural validity assessment**: Evaluating how accurately and respectfully the platform represents traditional knowledge from the perspective of knowledge-holding communities.
- **User experience evaluation**: Assessing usability, engagement and satisfaction among diverse user groups.
- **Outcome measurement**: Measuring the platform’s impact on dietary behavior, nutritional knowledge, cultural connection and health outcomes where applicable.
- **Systematic refinement**: Using evaluation results to continuously improve both cultural representation and technical functionality.
- **Knowledge expansion**: Establishing processes for incorporating additional traditional knowledge and practices over time.

This phase ensures that the integration process remains responsive to both community needs and practical outcomes, supporting ongoing improvement rather than static implementation.

Importantly, the outcomes of Phase 4 do not only inform future iterations of the platform but actively feed back into earlier phases of the model. Cultural validity assessments and user feedback may reveal gaps in the original documentation (Phase 1), prompting deeper or more inclusive community engagement. Similarly, the evaluation of user experience and system performance can highlight the need for revised ontologies, data structures or interface designs in Phase 2. Even implementation strategies (Phase 3) may require adjustment to improve governance models or ensure greater accessibility across user groups. By creating intentional feedback loops into all previous phases, the model supports a dynamic and evolving integration process—one that prioritizes continuous learning, community accountability and cultural integrity at every stage of digital development.

### 7.2. Implementation Considerations

The successful implementation of this framework requires attention to several critical factors that cut across all phases:

#### 7.2.1. Resource Requirements

Proper integration of traditional nutritional knowledge into digital platforms requires significant resources, including the following:

- **Time**: Relationship-building with knowledge-holding communities and participatory processes require extended timeframes beyond conventional development cycles.
- **Financial resources**: Comprehensive documentation, custom technical development and ongoing community engagement require substantial funding.
- **Expertise**: Successful implementation requires interdisciplinary teams including cultural anthropologists, nutritionists, software developers, user experience designers and community representatives.
- **Technical infrastructure**: Flexible platforms capable of representing complex traditional knowledge may require more sophisticated technical infrastructure than conventional nutrition applications.

Organizations should realistically assess these requirements before initiating integration projects to ensure sustainability throughout the process.

#### 7.2.2. Scalability Approaches

While deep cultural integration often starts with specific traditional food systems, several approaches can support scaling across multiple traditions:

- **Modular architecture**: Designing platforms with modular components that can accommodate multiple traditional systems through customization rather than complete redesign.
- **Common ontology frameworks**: Developing flexible ontology frameworks that can represent diverse traditional systems while maintaining their unique characteristics.
- **Knowledge commons**: Establishing shared repositories of traditional food information with appropriate governance and access protocols.
- **Open standards**: Developing open technical standards for representing traditional food knowledge that can be implemented across multiple platforms.

These approaches can help balance the depth required for authentic representation with the breadth needed for practical impact.

#### 7.2.3. Sustainability Strategies

The long-term sustainability of traditional knowledge integration requires attention to the following:

- **Economic models**: Developing sustainable business models that support ongoing operations while respecting cultural ownership of traditional knowledge.
- **Knowledge maintenance**: Establishing processes for updating and expanding traditional knowledge over time as practices evolve.
- **Community capacity building**: Building technical and governance capacity within knowledge-holding communities to maintain their agency in digital representations.
- **Institutional partnerships**: Forming long-term partnerships with research institutions, healthcare systems and cultural organizations to support ongoing development.

These sustainability considerations should be addressed from the earliest stages of project planning rather than as afterthoughts once implementation is complete.

## 8. Ethical Considerations

Integrating traditional nutritional knowledge into digital platforms raises important ethical considerations that extend beyond technical implementation. These considerations must be centered in all phases of development to ensure respectful and beneficial outcomes.

### 8.1. Intellectual Property and Attribution

Traditional knowledge often exists in collective cultural contexts that don’t align with conventional intellectual property frameworks designed for individual innovation [71]. Digital platforms must navigate these tensions through approaches that

- Recognize collective rather than individual ownership where appropriate;
- Implement clear attribution mechanisms that acknowledge communities of origin;
- Respect restrictions on use that may accompany traditional knowledge;
- Develop benefit-sharing models that return value to knowledge-holding communities.

Research on ethical frameworks for traditional knowledge digitization suggests that conventional copyright or patent approaches are often inadequate, requiring instead customized arrangements that reflect specific cultural contexts and community wishes [72]. Digital nutrition platforms incorporating traditional knowledge should develop explicit policies addressing these issues in consultation with affected communities.

### 8.2. Inclusion of Religious Dietary Systems

In addition to respecting the cultural origins of traditional nutritional knowledge, ethical integration must also accommodate religious dietary frameworks that shape food choices in many communities. Faith-based dietary rules, such as halal, kosher or fasting guidelines during Ramadan or Lent, are deeply embedded in cultural identity and well-being. Their omission from digital nutrition platforms risks both cultural insensitivity and functional irrelevance for large populations. Therefore, ethical database design should ensure the accurate representation of these dietary systems, including religious classifications, preparation methods and eventually restrictions. Incorporating religious dietary frameworks into culturally adaptive nutrition platforms not only enhances inclusivity but also fosters trust and user engagement across diverse spiritual contexts [73].

### 8.3. Informed Consent and Governance

Meaningful informed consent for digitizing traditional knowledge requires processes that

- Provide full transparency about how knowledge will be used, displayed and shared;
- Communicate in culturally appropriate formats and languages;
- Respect community decision-making structures and timeframes;
- Acknowledge that different communities may have different standards for what constitutes appropriate consent.

Traditional knowledge is often held collectively rather than individually, requiring consent processes that engage with legitimate community governance structures [72]. Digital platforms must navigate complex questions about who has the authority to grant permission for knowledge sharing and how ongoing consent can be maintained over time.

Research on traditional knowledge governance suggests that effective approaches typically involve multiple layers of governance, including (1) community-level protocols that establish local standards for knowledge sharing; (2) platform-level policies that implement these protocols in digital contexts; and (3) external oversight mechanisms that ensure compliance with both community protocols and broader ethical standards [74].

These governance approaches must be built into digital platforms from the earliest stages of development rather than added as afterthoughts. Platforms that establish clear governance structures from the outset tend to achieve higher levels of community trust and more sustainable knowledge-sharing relationships.

### 8.4. Digital Equity and Access Considerations

While digital platforms offer powerful tools for preserving and sharing traditional nutritional knowledge, they also raise important questions about who can access and benefit from these resources. Digital divides—including disparities in internet access, device availability, digital literacy and language support—may limit participation precisely among the communities whose knowledge is being digitized [75].

Addressing these equity concerns requires attention to the following:

- **Infrastructure access:** Ensuring that knowledge-holding communities have the technical infrastructure necessary to access and participate in digital platforms.
- **Capacity building:** Supporting digital literacy development within traditional communities through training, education and technical support.
- **Multilingual approaches:** Developing platforms that support indigenous and minority languages rather than requiring translation into dominant languages.
- **Culturally appropriate interfaces:** Designing user experiences that align with diverse cultural communication patterns and technology relationships.
- **Offline functionality:** Creating systems that can function in contexts with limited or intermittent connectivity.

Without explicit attention to these equity dimensions, digital integration efforts risk creating two-tiered systems where traditional knowledge becomes more accessible to external researchers and commercial entities than to the communities from which it originates [76]. Ethical integration requires designing for equitable access from the outset, with particular attention to the needs and constraints of knowledge-holding communities.

## 9. Conclusions and Future Directions

The integration of traditional nutritional wisdom into digital health platforms represents both a significant opportunity and a complex challenge. This perspective has outlined the value of traditional food systems for contemporary health, identified barriers to digital adaptation, examined promising integration models and proposed a structured framework for future development.

### 9.1. Summary of Key Findings

Our analysis suggests several important conclusions:

1. **Traditional nutritional wisdom offers significant value** for contemporary health promotion through its holistic frameworks, cultural relevance and proven health benefits. These systems provide a wealth of knowledge about sustainable, culturally appropriate dietary patterns that can enhance the effectiveness of digital nutrition platforms.
2. **Current digital platforms demonstrate significant limitations** in cultural inclusivity and traditional knowledge integration. Standardization around Western nutritional concepts, limited representation of cultural context and insufficient adaptability to diverse food systems represent missed opportunities for more personalized and relevant nutrition guidance.
3. **Technical and cultural barriers to integration are substantial** but not insurmountable. While challenges exist in data architecture, algorithm design, cultural representation and ethical implementation, successful case studies demonstrate that thoughtful approaches can effectively bridge traditional and digital contexts.
4. **The proposed four-phase integration framework**—documentation, digital adaptation, implementation and evaluation—provides a structured approach for researchers, developers and communities seeking to incorporate traditional nutritional wisdom into digital platforms in ways that maintain cultural integrity while harnessing technological capabilities.
5. **Ethical considerations must be centered** throughout the integration process, with particular attention to knowledge protection, informed consent, governance mechanisms and digital equity. These ethical dimensions are not peripheral concerns but foundational requirements for successful integration.

### 9.2. Implications for Research and Practice

These findings have significant implications for multiple stakeholders:

For **health technology developers**, our analysis emphasizes the importance of moving beyond standardized nutritional models to incorporate greater cultural diversity in digital platforms. This will likely require a fundamental rethinking of data architectures, algorithm design and user interfaces to accommodate traditional knowledge systems.

For **healthcare providers**, the integration of traditional nutritional wisdom into digital tools offers opportunities for more culturally responsive care, particularly in diverse populations where standard nutritional guidance may lack resonance or cultural relevance.

For **traditional knowledge-holding communities**, digital platforms represent potential tools for preserving and revitalizing food traditions while ensuring that these traditions inform contemporary nutrition approaches. However, careful attention to governance and equity is essential to ensure that these communities maintain control over their knowledge.

For **researchers**, this perspective highlights the need for interdisciplinary approaches that bridge nutrition science, cultural anthropology, computer science and implementation research. Future studies should evaluate the effectiveness of culturally adapted digital nutrition interventions and refine methodologies for ethical knowledge integration.

### 9.3. Future Research Directions

Building on this perspective, several key areas warrant further research:

1. **Standardized evaluation frameworks** for measuring the cultural authenticity and effectiveness of digital platforms that incorporate traditional nutritional knowledge. Such frameworks would enable more systematic comparison across different integration approaches.
2. **Technical innovation in knowledge representation**, including exploration of semantic web technologies, ontology development and machine learning approaches that can better accommodate the relational and contextual nature of traditional food systems.
3. **Implementation science research:** examining factors that influence the successful uptake and sustained use of culturally adapted digital nutrition platforms in diverse contexts.
4. **Participatory design methodologies** specifically tailored for working with traditional knowledge systems, with attention to power dynamics, cultural protocols and diverse ways of knowing.
5. **Policy frameworks** for protecting traditional knowledge in digital contexts, including the development of guidelines, standards and regulatory approaches that support ethical integration.

While global integration is desirable, we argue that a hybrid model—linking locally governed, culturally specific databases to broader, interoperable frameworks—may offer the most effective path forward. These community-rooted repositories can ensure contextual accuracy and cultural control, while still contributing to a larger network that enables global access and cross-cultural learning.

The integration of traditional nutritional wisdom into digital platforms represents a vital opportunity to enhance the cultural relevance, effectiveness and inclusiveness of nutrition technology. By bridging ancient wisdom with digital innovation through thoughtful, ethical and community-centered approaches, we can develop nutrition tools that honor cultural diversity while addressing contemporary health challenges. As digital health technologies continue to evolve, ensuring that they represent and respect diverse nutritional traditions will be essential for truly personalized and culturally responsive approaches to health promotion.

Generative artificial intelligence (ChatGPT 4o, OpenAI) was used to assist with language refinement and structural editing throughout the manuscript. No AI tools were used for data generation, analysis or study design.

## Figures and Tables

**Table 1:** Cultural inclusivity assessment of popular digital nutrition platforms.

Platform	Food Database Diversity	Cultural Context	Traditional Principles	Adaptability
MyFitnessPal	Medium (primarily Western-centric)	Low	None	Low
Cronometer	Medium–High	Low	None	Medium
Noom	Medium	Medium	Limited	Medium
Lifesum	Medium	Low	Limited	Low
Nutritics	High	Medium	None	Medium

Assessment based on platform analysis conducted January–March 2025.
